# Combined Industrial Wastewater Treatment in Anaerobic Bioreactor Posttreated in Constructed Wetland

**DOI:** 10.1155/2013/957853

**Published:** 2013-12-16

**Authors:** Bibi Saima Zeb, Qaisar Mahmood, Saima Jadoon, Arshid Pervez, Muhammad Irshad, Muhammad Bilal, Zulfiqar Ahmad Bhatti

**Affiliations:** ^1^Department of Environmental Sciences, COMSATS Institute of Information Technology, Abbottabad 22060, Pakistan; ^2^Department of Natural Resources Engineering and Management, University of Kurdistan, Hewler, Iraq

## Abstract

Constructed wetland (CW) with monoculture of *Arundo donax* L. was investigated for the posttreatment of anaerobic bioreactor (ABR) treating combined industrial wastewater. Different dilutions of combined industrial wastewater (20, 40, 60, and 80) and original wastewater were fed into the ABR and then posttreated by the laboratory scale CW. The respective removal efficiencies of COD, BOD, TSS, nitrates, and ammonia were 80%, 78–82%, 91.7%, 88–92%, and 100% for original industrial wastewater treated in ABR. ABR was efficient in the removal of Ni, Pb, and Cd with removal efficiencies in the order of Cd (2.7%) > Ni (79%) > Pb (85%). Posttreatment of the ABR treated effluent was carried out in lab scale CW containing *A. donax* L. CW was effective in the removal of COD and various heavy metals present in ABR effluents. The posttreatment in CW resulted in reducing the metal concentrations to 1.95 mg/L, 0 mg/L, and 0.004 mg/L for Ni, Pb, and Cd which were within the permissible water quality standards for industrial effluents. The treatment strategy was effective and sustainable for the treatment of combined industrial wastewater.

## 1. Introduction 

Pakistan's current population of 180 million is expected to grow to about 221 million by the end of year 2025 [1]. In Pakistan and other developing countries, water pollution is a major threat to the livelihood of people [2]. The heavy metals contamination of aquatic and terrestrial ecosystems is a major environmental problem. Each pollution problem calls for specific optimal and cost-effective solution; if one technology proves less or ineffective, the other takes its place. It is indispensable to treat industrial wastewaters for their subsequent use for irrigation, drinking, and other purposes. In addition, due to an increased scarcity of clean water, there is a need for appropriate management of available water resources [3]. The factors like profound demographic, economic changes and global energy crisis are compelling the implementation of low-cost natural treatment systems for the domestic and industrial wastewaters [1].

In the recent years, the wastewater treatment strategies have been shifted to one of the most promising methods, that is, biological anaerobic treatment with the adoption of high rate anaerobic systems like upflow anaerobic sludge blanket (UASB) and other related treatment systems. The outstanding characteristics of high rate ABR include the anaerobic microorganisms capable of aggregation, low operational and maintenance costs, energy recovery in the form of biogas, low energy consumption, and low production of digested sludge [4–7]. In developing countries like India, Brazil, and Colombia, where financial resources are generally scarce due to high energy costs, the process is familiar as one of the most feasible methods for the wastewater treatment. Despite several modifications, the quality of ABR treated effluent hardly ever meets the discharge standards [6, 8]. Lettinga and coresearchers applied ABR process for municipal wastewater treatment since early 1980 [9–13] and reported that about 70% of chemical oxygen demand (COD) removal can be achieved under warm climates [6, 14, 15]. Since its inception, wider hype has been gained by this process [16, 17]. The ABR treated effluents can be employed for irrigation of various crops. However, such type of effluent may be high in chemical oxygen demand (COD), biochemical oxygen demand (BOD), and coliforms [18]. Thus, additional posttreatment strategy is mandatory for the ABR treated effluents if further use is desired [19–21].

CW wastewater treatment systems are engineered structures specifically designed for treating wastewater by optimizing the physical, chemical, and biological processes that occur in natural wetland ecosystems [1, 18, 22–24]. CW is known as green technology which uses plants for the removal of contaminants from a specified area, and process is known as phytoremediation [25]. CW is a low-cost or economical on-site wastewater treatment technology which is not only effective but also aesthetically pleasing. Since 1980 the utilization of the CW for the treatment of variety of wastewater has quickly become widespread. The amount of nutrients removed by plants and stored in their tissues is highly relative which depends on the plant type, biomass, and nutrient concentration in tissues [26].

The plant species, media like sand and gravel of specific ratio and size, and containers are the foundation materials for CW. There are two major types of CW, subsurface flowing water (SSF) CW and free water surface (FWS) flowing CW. A variety of macrophytes are used in CW and most common are floating macrophytes (i.e., *Lemna *spp. or *Eichhornia crassipes*), submerged macrophytes (i.e., *Elodea canadensis*), and rooted emergent macrophytes (i.e., *Phragmites australis* and *Typha angustifolia*). The plants roots create conducive environment for the microbial growth and in winter the plant litter acts as insulator. CW is attached growth biological reactors, which tender higher pollutant removal efficiency through physical, chemical, and biological mechanisms. The common removal mechanisms associated with wetlands include sedimentation, coagulation, adsorption, filtration, biological uptake, and microbial transformation [3, 24, 27].

CW technology is well known at present, but it is not well documented for treating specific industrial effluents [28–30]. A variety of posttreatment configurations based on various combinations with ABR have been studied; ABR followed by final polishing units (FPU) or polishing pond (PP) is a common process used in India, Colombia, and Brazil due to its simplicity in operation [6, 31–33]. The implementation of low-cost, simple mitigation measures is required for the timely control and sustainable management of pollution problems in developing countries. The objective of this study was to evaluate the performance of ABR for the treatment of combined industrial wastewater followed by posttreatment in CW planted with *A. donax.*

## 2. Materials and Methods

### 2.1. Collection of Wastewater and Treatment

The industrial wastewater was collected from combined drain at Hattar Industrial Estate, Hattar, Pakistan, as grab samples. The physicochemical parameters like pH, turbidity, and EC were determined onsite while the rest were analyzed in the laboratory within 24 h. As a treatment strategy and to avoid toxic effects of the pollutants, various dilutions of wastewater included 20, 40, 60, and 80% to feed into ABR, after which original wastewater was also treated in ABR and then CW.

### 2.2. ABR Experimental Setup

**Figure 1:**
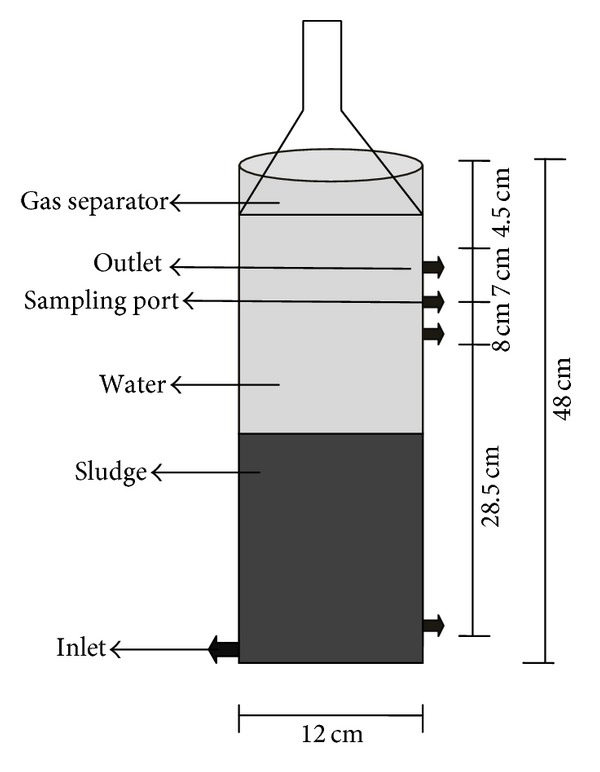
Schematic diagram of a lab scale anaerobic bioreactor with its dimensions.

This research work was carried out in the bioremediation laboratory of COMSATS Institute of Information Technology, Abbottabad, Pakistan. In this study ABR was used as a primary treatment step. A lab scale ABR was operated in upflow mode with biomass retention as shown in Figure 1. The reactor is made of Perspex with a working volume of 5 liters. The influent was pumped into ABR using peristaltic pump from the influent vessel to the reactor (Figure 1). The flow rate was adjusted according to results of startup study. A recycling pump was used to mix the influent (substrate) and sludge (biocatalyst) and to decrease possible substrate inhibition. The ratio of recycle flow to the influent flow was set at about 2.5–3. Bioreactor startup was carried out by feeding synthetic wastewater and nutrient solution at various organic loading rate (OLR) and COD by using organic compounds, at a fixed Hydraulic retention time (HRT) but increasing OLR and at fixed OLR but decreasing HRT.

Industrial wastewater samples were collected from Hattar Industrial Estate (Haripur, Pakistan) after characterization and were fed into the ABR. Different dilutions of real industrial wastewater were made to avoid the reactor disturbances before feeding to ABR.

### 2.3. Experimental Setup

**Figure 2:**
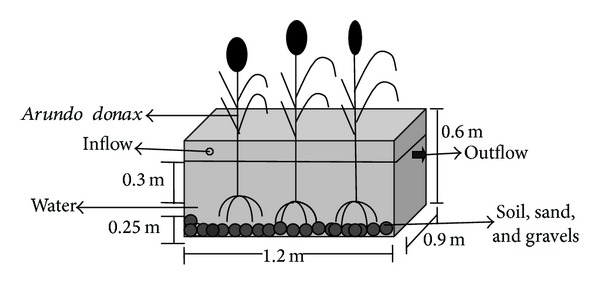
Schematic diagram of a laboratory scale CW for the treatment of combined industrial wastewater.

The lab scale experimental constructed wetland consists of two independent rectangular basins (length: 120 cm, width: 90 cm, and depth: 40 cm). The basins were filled with gravel, sand, and soil from bottom to top with one layer of each as shown in Figure 2. Each basin had a 10% slope and was equipped with a nozzle outlet for discharging the treated effluent. The CW was planted with *A. donax* (6 shoots/m²) taken from the botanical garden of the institute.

An unplanted bed served as a control. Treated effluents were collected directly from the lab scale experimental plant of the wastewater treatment laboratory. The operational conditions of the experimental setup of CW are shown in Table 1. All plants, sand, and gravel were properly washed before planting to CW system.

Pollutant removal rates (%) were calculated according to the following equation:

(1) $$\begin{array}{c} R\;\left(\%\right)=\left[1-\left(\frac{Cf}{Ci}\right)\right]\times 100, \end{array}$$

where *R* is the removal rate, *Ci* is the concentration (mg/L) of the considered parameter in the untreated WW (influent), and *Cf* is the concentration (mg/L) of the considered parameter in the treatment bed effluent.

### 2.4. Analytical Procedures

Raw and treated samples were analyzed for their BOD, COD, EC, pH, turbidity, and so forth, according to the standard methods [34]. For COD determination closed reflux, calorimetric method included digestion at 150°C for 2 h in COD vials followed by spectrophotometer reading at 530 nm [34]. The pH was measured using a digital pH meter (HANNA, HI 991003 Sensor Check pH) and TDS and conductivity by HANNA, HI9835 Microprocessor for conductivity and TDS. Heavy metals were analyzed through atomic absorption spectrophotometer. At least three readings were taken for each parameter each time and then mean value was calculated.

### 2.5. Statistical Analysis

Collected data were analyzed by the descriptive statistics and arithmetic averages of percent removal were calculated using Microsoft Excel XP version 2010 and Origin Lab 8.

## 3. Results and Discussion

### 3.1. Characterization of Combined Industrial Wastewater

Physicochemical characteristics of industrial wastewater were depicted in Table 2. Four different dilutions (20%, 40%, 60%, and 80%) and original WW from HIE had the following characteristics.

### 3.2. Pretreatment of Combined Industrial Wastewater in ABR

The ABR was fed with combined industrial wastewater for treatment at retention time of 12 h. The treated effluent characteristics and percent removal efficiency was showed in Table 3.

**Figure 3:**
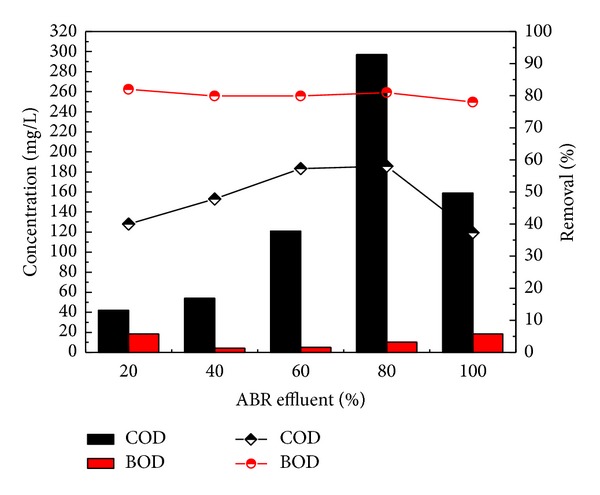
COD removal efficiency of a lab scale ABR reactor for combined industrial wastewater.

The results described the performance of the ABR for the treatment of combined industrial wastewater, as the concentrations of COD before pretreatment were 70, 189, 284, 379, and 474 mg/L, respectively, for four different dilutions of 20, 40, 60, and 80 and original wastewater. After pretreatment with ABR the COD was reduced to 42, 54, 121, 159, and 297 mg/L with 40.0, 40.8, 57.3, 58.0, and 37.3% removal efficiency, respectively. The results in Figure 3 showed the maximum COD removal efficiency for the 80% dilution of the wastewater through ABR. The ABR also reduced the BOD concentrations of the dilutions from 78 to 82% as shown in Figure 3. The BOD concentration reduced from 23.3, 25.4, 50.9, 77.0 and 84.8 mg/L to 18.5, 4.16, 5.1, 10.2, and 18.5, respectively. It was observed from the results that ABR showed excellent removal efficiency for BOD removal.

**Figure 4:**
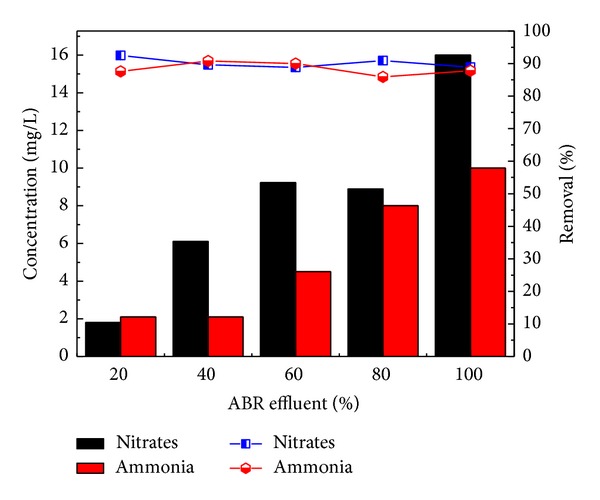
Nitrates Removal efficiency of pretreatment of combined industrial wastewater with ABR.

Total solids were tremendously removed by 91.7% with the corresponding concentration of 1400 mg/L for original wastewater. The concentration of NO_3_-N was reduced from 24, 59, 83, 98, and 145 to 1.8, 6.1, 9.23, 8.9, and 16 mg/L for 20, 40, 60, and 80 and original wastewater. ABR showed 88 to 92% removal efficiency for the NO_3_-nitrogen as shown in Figure 4.

**Figure 5:**
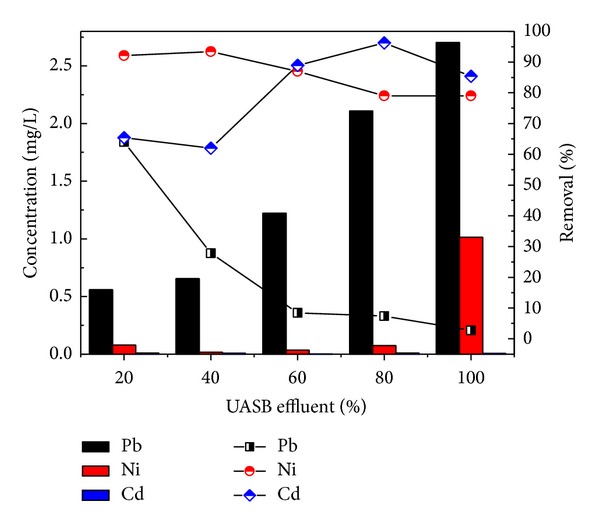
Removal efficiency of Pb, Ni, and Cd by ABR.

Similarly, the removal efficiency of NH_4_-N was 87.6, 90.8, 90, 85.9, and 87.8% for the four different dilutions 20, 40, 60, and 80 and original wastewater, respectively, as shown in Figure 4. The concentrations of NH_4_-N 17, 23, 45, 57, and 82 mg/L were reduced to 2.1, 2.1, 4.5, 8, and 10 mg/L. On the other hand, Pb, Ni, and Cd removal by reactor was 2.7%, 79%, and 85% for real industrial wastewater. The heavy metals removal were found in order Cd > Ni > Pb shown in Figure 5.

The previous workers observed that the treatment of complex industrial wastewater reduced the efficiency of the ABR as in the present study [35]. During anaerobic digestion processes of organic matter the biochemical reaction takes place which is affected by the heavy metals presence [35]. It is clear from the results that soluble heavy metals rapidly decreased at the initial concentrations. It depends on which chemical form the heavy metal is existed. The most common and important form of the heavy metals are precipitation (as sulfides, carbonates, and hydrocarbons) and sorption on to solid form (Inhibition effect of heavy metals on anaerobic sludge) [36]. Ni could be bound in all forms. So it was cleared that high initial concentrations were tolerated by the ABR sludge and thus showed the satisfactory removal of the heavy metals.

However, the residual concentration of organic (BOD and COD) and heavy metals in the anaerobic reactor effluent usually exceeds the maximum permissible level prescribed by the effluent discharge standards of most developing countries [20, 37, 38]. From this standpoint, posttreatment of anaerobic effluent is necessary to reduce these contaminants to the required level [39].

### 3.3. Posttreatment of ABR Effluent with Constructed Wetland

**Figure 6:**
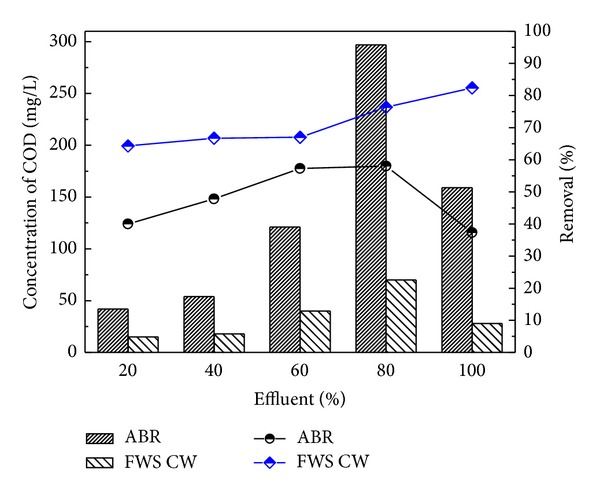
Comparison of ABR and CW for COD removal.

**Figure 7:**
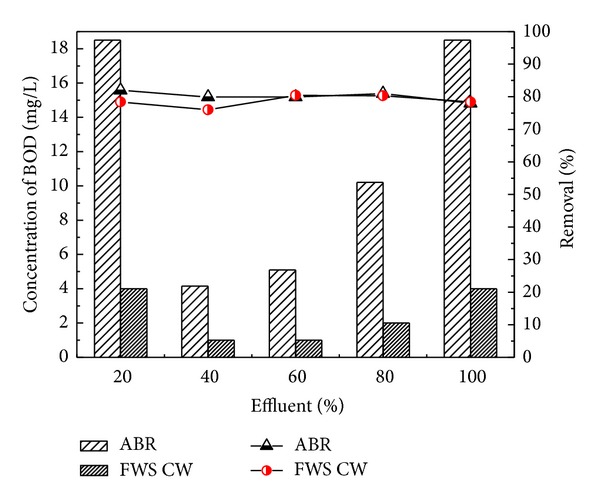
Comparison of ABR and CW for BOD removal.

**Figure 8:**
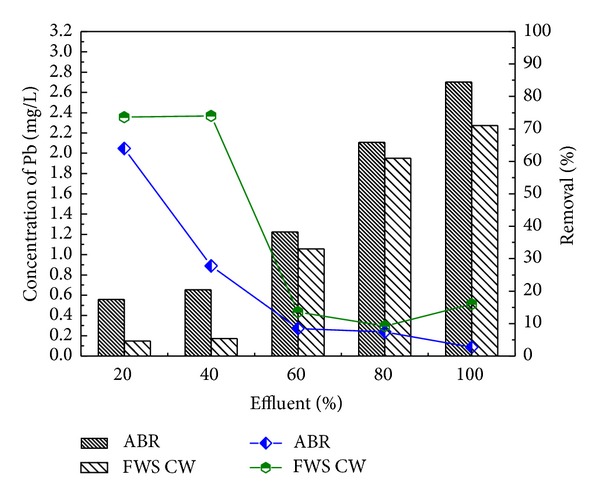
Comparison of % removal efficiency of Pb in ABR and FWS CW.

**Figure 9:**
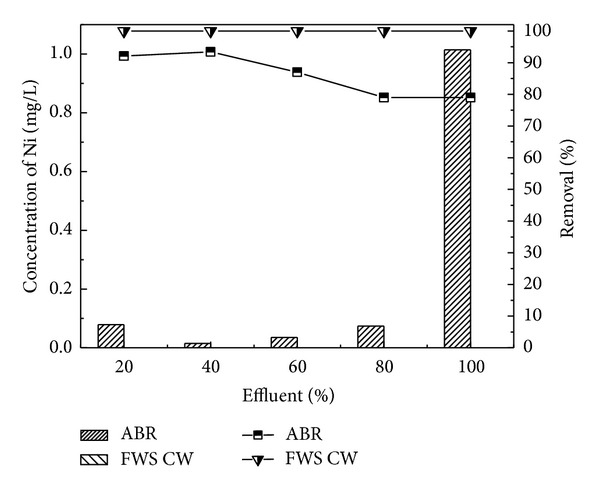
Comparison of percent removal efficiency of Ni in ABR and FWS SCW.

**Figure 10:**
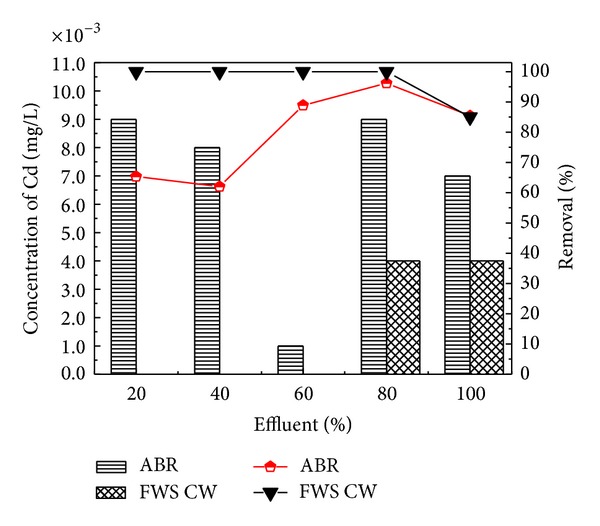
Comparison of % removal efficiency of Cd in ABR and FWS CW.

The pretreated effluent was then further treated by CW at HRT of 3 days for each dilution. The results for FWS CW effluent are shown in Figures 6, 7, 8, 9, and 10 with pollutant percent removal efficiency.

The results of treatment in CW showed efficient removal efficiency for COD, BOD, TS, nitrates, ammonia, and heavy metals like Pb, Ni, and Cd. The residual concentration of COD and BOD was 64.3, 66.7, 67, 76.4, and 82.4 and 78.4, 76, 80.3, 80.3, and 78.4 mg/L, respectively, for the corresponding dilutions of 20, 40, 60, and 80 and original WW as shown in Figures 6 and 7. The CW showed the highest COD removal efficiency of 82.4% for original WW, but at the same dilution the BOD was reduced to 78.4%. Nitrates and ammonia removal efficiency was found to be 95, 82, 86, 72, and 75% for the respective concentrations of 1.8, 6.1, 9.23, 8.9, and 16 mg/L of the corresponding four different dilutions and original pretreated effluent. Ammonia removal was not satisfactory as compared to other parameters and the highest removal efficiency was 70.1% by CW.

CW normally improves the DO in wetland. The introduction of excess organic matter may result in a depletion of oxygen from an aquatic system. Prolonged exposure to low dissolved oxygen levels (<5.0-6.0 mg/L) may not directly kill an organism but will increase its susceptibility to other environmental stresses. Exposure to <30% saturation (<2.0 mg/L oxygen) for one to four days kills most of the biota in a system. If oxygen-requiring organisms perish, the remaining organisms will be air-breathing insects and anaerobic (not requiring oxygen) bacteria [40]. If all oxygen is depleted, aerobic (oxygen-consuming) decomposition ceases. So, treating pollutants in wetlands may help to increase DO which is consumed by the other aerobes. In this experiment during the posttreatment by CW the DO increased up to 8.8 mg/L.

Industrial wastewater was treated in two-stage constructed wetland [41] planted with *Typha latifolia* and *Phragmites australis*. For tannery wastewater, CW may be an interesting treatment option. Two-stage series of horizontal subsurface flow CW with *Phragmites australis* (UP series) and *Typha latifolia *(UT series) provided high removal of organics from tannery wastewater, up to 88% of biochemical oxygen demand (BOD_(5)_) (from an inlet of 420 to 1000 mg/L) and 92% of chemical oxygen demand (COD) (from an inlet of 808 to 2449 mg/L) and of other contaminants, such as nitrogen, operating at hydraulic retention times of 2, 5, and 7 days. Overall mass removals of up to 1294 kg COD/ha/d and 529 kg BOD_(5)_/ha/d were achieved for a loading ranging from 242 to 1925 kg COD ha/d and from 126 to 900 kg BOD_(5)_/ha/d. Plants were resilient to the conditions imposed; however *P. australis* exceeded *T. latifolia* in terms of propagation. In the present study, *A. donax* was used in the CW for post treatment which showed the efficient performance for the further removal of pollutants from the ABR pretreated effluent. The results confirmed that effluent showed traces of heavy metals Ni and Cd with the corresponding ABR treated wastewater at almost all the levels of dilutions of 20, 40, 60, and 80 and original wastewater. CW showed the maximum removal efficiency for Ni and Cd as depicted in Figures 9 and 10, respectively. CW posttreatment of Pb was not satisfactory in the reduction of its concentration. Using the San Joaquin Marsh constructed wetlands, the removal efficiencies for four heavy metal elements Cd, Cu, Pb, and Zn were evaluated. It was found that the effluent metal concentrations were not substantially lower than the influent. The removal efficiencies of 23.9%, 10.6%, and 17.6% were found for Cd, Cu, and Zn, respectively. No significant reduction was observed for concentrations of Pb [42].

Metal and metalloid removal in constructed wetlands from industrial waste water have been investigated [43]. The removal of metals and metalloids from contaminated waters was investigated in constructed wetlands. Metal removal rates in wetlands depend on the type of element (Hg > Mn > Fe 1/4 Cd >Pb 1/4 Cr > Zn 1/4 Cu > Al > Ni > As), their ionic forms, substrate conditions, season, and plant species. Standardized procedures and data are lacking for efficiently comparing properties of plants and substrates. The study depicted the relative treatment efficiency index (RTEI) to quantify treatment impacts on metal removal in constructed wetlands.

Various mechanisms, including sedimentation, filtration, chemical precipitation, adsorption, microbial interactions, and uptake by vegetation, have been attributed with the removal of metal within CW. Specifically, the major processes that are responsible for metal removal in CW are binding to sediments and soils, precipitation as insoluble salts, and uptake by plants and bacteria [44]. In CW, substrate interactions remove most metals from contaminated water [45]. The anoxic condition of wetland soil helps create an environment for immobilization of heavy metals in the highly reduced sulfite or metallic form [46]. Wetland plants adsorb and accumulate metals in tissues, which can play an important role in CW pollutant treatment efficiency [47]. Phytoremediation, using vegetation to remove, detoxify, or stabilize heavy metal pollutants, is an accepted tool for cleaning polluted soils and waters [48]. Research has also shown that metal storage in the sediment is influenced by vegetation. Concentrations of metals were significantly higher in the vegetated sediments than in the nonvegetated sediments [49].

## 4. Conclusion

This paper presented the evaluation results on removal efficiencies for COD, BOD, nitrates, phosphates, TS, and heavy metals (Cd, Ni, and Pb) in ABR and posttreated by a lab scale *Arundo donax* based CW. It was clearly observed that posttreatment accomplished tremendous removal of the COD, BOD, TS Ni, and Cd. The efficiency of both the treatment systems was not too much satisfactory for Pb removal. Because of the positive effects of vegetation on metal removal efficiency, CWs containing *A. donax* is recommended for HIE combined wastewater treatment.

## Figures and Tables

**Table 1:** Dimensions and operating conditions of experimental CW.

Dimensions of CW	
Length	1.2 m
Width	0.9 m
Total height	0.6 m
Total container volume	0.432 m^3^
Water depth	0.3 m
Substrate depth	0.25 m
Plant name	*Arundo donax* L. (giant reed)
No. of rhizome/m^2^	3
Operating conditions	
OLR	538 kg/ha/d
HRT	3 days
HLR	862 m^3^/ha/d

**Table 2:** Characterization of four different dilutions and original WW of combined industrial wastewater.

Parameters	Influent/raw wastewater dilutions				
	20%	40%	60%	80%	Original WW
pH	8.1	8.48	8.6	8.76	10.2
Conductivity (*µ*s)	627	646	645	676	702
TDS	378	330	344	394	411
TS	712	812	780	1333	1400
VS	600	520	1050	1160	1960
COD	70	189	284	379	474
BOD	23.3	25.4	50.9	77	84.8
Nitrates	24	59	83	98	145
Ammonia	17	23	45	57	82
Lead	0.131	0.653	1.335	1.851	2.337
Nickel	0.117	0.225	0.315	0.271	0.403
Cadmium	0.026	0.021	0.009	0.026	0.048

The values are given as mg/L except pH and conductivity.

**Table 3:** The performance of ABR in treating combined industrial effluents.

Parameters	Influent/raw wastewater					ABR effluent					% removal				
	20%	40%	60%	80%	Original WW	20%	40%	60%	80%	Original WW	20%	40%	60%	80%	Original WW
pH	8.1	8.48	8.6	8.76	10.2	7.89	8.31	8.52	8.66	8.41	—	—	—	—	—
Conductivity (*µ*s)	627	646	645	676	702	654	616	645	687	702	—	—	—	—	—
TDS	378	330	344	394	411	339	324	334	357	365	10	2	3	9	11
TS	712	812	780	1333	1400	83	85	93	215	115	88	89.5	88	83.8	91.7
VS	600	520	1050	1160	1960	45	86.3	163.9	97.2	305	92	83.5	86	90	84
COD	70	189	284	379	474	42	54	121	159	297	40	47.8	57.3	58.04	37.34
BOD	23.3	25.4	50.9	77	84.8	18.5	4.16	5.1	10.2	18.5	82	80	80	81	78
Nitrates	24	59	83	98	145	1.8	6.1	9.23	8.9	16	92.5	89.6	88.8	90.9	88.9
Ammonia	17	23	45	57	82	2.1	2.1	4.5	8	10	87.6	90.8	90	85.9	87.8
Lead	0.131	0.653	1.335	1.851	2.337	0.558	0.653	1.222	2.109	2.703	64	27.8	8.46	7.4	2.7
Nickle	0.117	0.225	0.315	0.271	0.403	0.079	0.015	0.035	0.074	1.014	92.1	93.4	87	79	79
Cadmium	0.026	0.021	0.009	0.026	0.048	0.009	0.008	0.001	0.009	0.007	65.4	62	88.9	96.2	85.4

The values are given as mg/L except pH and conductivity.
