# Corrigendum to “Combined Industrial Wastewater Treatment in Anaerobic Bioreactor Post-Treated in Constructed Wetland”

**DOI:** 10.1155/2020/6102379

**Published:** 2020-07-24

**Authors:** Bibi Saima Zeb, Qaisar Mahmood, Saima Jadoon, Arshid Pervez, Muhammad Irshad, Muhammad Bilal, Zulfiqar Ahmad Bhatti

**Affiliations:** ^1^Department of Environmental Sciences, COMSATS Institute of Information Technology, Abbottabad 22060, Pakistan; ^2^Department of Natural Resources Engineering and Management, University of Kurdistan, Erbil, Iraq

In the article titled “Combined Industrial Wastewater Treatment in Anaerobic Bioreactor Post-treated in Constructed Wetland” [1], there were typographical errors and errors in the figures. These have been corrected in the revised version shown below:
The abstract was revised for typographical errorsLanguage corrections were made in the textThe objectives of the study were clearly mentionedAll acronyms were spelled out when used for the first timeCorrections were made to the captions of Figures 1 to 10, to aid clear understanding of the plotsTable 2 was deleted as its values were repeated in Table 3 and hence section 3.1 was also deletedA separate Discussion section was addedAn additional article was cited as reference 42 [2] and the previous references 42-49 became references 43-50

The corrected article is as follows.


**Abstract**


Constructed Wetland (CW), containing monoculture of *Arundo donax* L., was investigated for the post-treatment of effluent from Anaerobic Bioreactor (ABR) treating combined industrial wastewater. Different dilutions of combined industrial wastewater (20, 40, 60, and 80% v/v) and original wastewater were fed into the ABR as pretreatment. ABR effluent was post-treated by the laboratory scale CW. The removal efficiencies of Chemical Oxygen Demand (COD), Biochemical Oxygen Demand (BOD), Total Suspended Solids (TSS), nitrates and ammonia were 80%, 78-82%, 91.7%, 88-92% and 100%, respectively, for original industrial wastewater treated in ABR. ABR was found efficient in the removal of the metals Ni, Pb and Cd with removal efficiencies in the order of Cd (2.7%) > Ni (79%) > Pb (85%). Post-treatment of the ABR-treated effluent was carried out in lab scale CW planted with *A. donax* L. CW was effective in removal of COD and various heavy metals present in ABR effluents. Post-treatment in CW resulted in reducing the metal concentrations of Ni, Pb and Cd to 1.95 mg/L, 0 mg/L and 0.004 mg/L, respectively, which were within the limits of permissible water quality standards for industrial effluents. The treatment strategy was effective and sustainable to treat combined industrial wastewater.

## 1. Introduction

Pakistan's current population of 180 million is expected to grow to about 221 million by the year end 2025 [1]. In Pakistan and other developing countries, water pollution is a major threat to the livelihood of people [2]. Heavy metal contamination of aquatic and terrestrial ecosystems is a major environmental problem. Each pollution problem calls for specific optimal and cost-effective solutions; if one technology proves less effective or ineffective, another takes its place. It is indispensable to treat industrial wastewaters for their subsequent use for irrigation, drinking and other purposes. In addition, due to an increased scarcity of clean water, there is a need for appropriate management of available water resources [3]. Factors like profound demographic changes, economic changes and the global energy crisis are compelling the implementation of low-cost natural treatment systems for domestic and industrial wastewaters [1].

In recent years, wastewater treatment strategies have shifted to one of the most promising methods, i.e. biological anaerobic treatment with the adoption of high rate anaerobic systems like up-flow anaerobic sludge blanket (UASB) reactor and other related treatment systems. The outstanding characteristics of high-rate Anaerobic Bioreactors (ABR) include anaerobic microorganisms capable of aggregation, low operational and maintenance costs, energy recovery in the form of biogas, low energy consumption and low production of digested sludge [4-7]. In developing countries like India, Brazil and Colombia, where financial resources are generally scarce due to high energy costs, the process is familiar as one of the most feasible methods for wastewater treatment. Despite several modifications, the quality of ABR-treated effluent hardly ever meets the discharge standards [6, 8]. Lettinga and co-researchers applied ABR process for municipal wastewater treatment since the early 1980s [9-13] and reported that about 70% chemical oxygen demand (COD) removal can be achieved under warm climates [6, 14, 15]. Since its inception, wider hype has been gained by this process [16, 17]. ABR-treated effluents can be employed for irrigation of various crops. However, such type of effluent may be high in COD, biological oxygen demand (BOD) and coliforms [18]. Thus, additional post-treatment strategy is mandatory for ABR-treated effluents if further use is desired [19-21].

CW wastewater treatment systems are engineered structures specifically designed for treating wastewater by optimizing the physical, chemical and biological processes that occur in natural wetland ecosystems [1, 18, 22-24]. CW is known as green technology, which uses plants for the removal of contaminants from a specified area, and the process is known as phytoremediation [25]. CW is a low cost or economical on-site wastewater treatment technology which is not only effective but also aesthetically pleasing. Since 1980, the utilization of the CW for the treatment of variety of wastewater has quickly become widespread. The amount of nutrients removed by plants and stored in their tissues is highly relative, which depends on the plant type, biomass and nutrient concentration in tissues [26].

The plant species, media like sand and gravel of specific ratio and size and containers are the foundation materials for CW. There are two major types of CW: subsurface flowing water (SSF) CW and free water surface (FWS) flowing CW. A variety of macrophytes are used in CW and the most common are floating macrophytes (i.e. *Lemna* spp or *Eichhornia crassipes*), submerged macrophytes (i.e. *Elodea canadiensis*) and rooted emergent macrophytes (i.e. *Phragmites australis* and *Typha anguistifolia*). The plants roots create a conducive environment for microbial growth and in winter the plant litter acts as an insulator. CW is an attached-growth biological reactor, which tender higher pollutant removal efficiency through physical, chemical and biological mechanisms. The common removal mechanisms associated with wetlands include sedimentation, coagulation, adsorption, filtration, biological uptake and microbial transformation [3, 24, 27].

CW technology is well-known at present, but it is not well documented for treating specific industrial effluents [28-30] and it can be used for polishing effluents of anaerobic bioreactors. A variety of post-treatment configurations based on various combinations with ABR have been studied; ABR followed by final polishing units (FPU) or polishing pond (PP) is a common process used in India, Colombia and Brazil due to its simplicity in operation [6, 31-33]. The implementation of low-cost, simple mitigation measures is required for the timely control and sustainable management of pollution problems in developing countries. The objective of this study was to evaluate the performance of ABR for the treatment of combined industrial wastewater followed by post-treatment in CW planted with *A. donax.*

## 2. Materials and Methods

### 2.1. Collection of Wastewater and Treatment

The industrial wastewater was collected from a combined drain at Hattar Industrial Estate (HIE), Hattar, Pakistan as grab samples. A number of industries are operating in HIE, like steel rerolling, paper recycling, ghee, food, beverages and cement. The physicochemical parameters like pH, turbidity and electric conductivity (EC) were determined onsite using portable pH, EC and turbidity analyzer, while the rest were analyzed in the laboratory within 24 h. As a treatment strategy and to avoid toxic effects of the pollutants, various dilutions (with distilled water) of wastewater included 20, 40, 60 and 80% (v/v) to feed into ABR, after which original wastewater was also treated in ABR and then CW.

### 2.2. ABR Experimental Set- up

This research work was carried out in the Bioremediation Laboratory of COMSATS Institute of Information Technology, Abbottabad, Pakistan. In this study, ABR was used as primary treatment step. A lab scale ABR was operated in up-flow mode with biomass retention as shown in Figure 1. The reactor is made of Perspex with a working volume of 5 L. The influent was pumped into ABR using a peristaltic pump from the influent vessel to the reactor (Figure 1). The flow rate was adjusted according to results of a startup study. A recycling pump was used to mix the influent (substrate) and sludge (biocatalyst) and to decrease possible substrate inhibition. The ratio of recycle flow to the influent flow was set at about 2.5-3. Bioreactor start up was carried out by feeding synthetic wastewater and nutrient solution at various Organic Loading Rate (OLR) and COD by using organic compounds at a fixed Hydraulic Retention Time (HRT) but increasing OLR, and at a fixed OLR but decreasing HRT. The ABR reactor in the Department of Environmental Sciences, COMSATS, Abbottabad was used for various types of the wastewater treatments in the past. Different wastewaters like domestic, industrial, municipal wastewater were treated through this ABR.

Inoculum was collected from the anaerobic methanogenic reactor in COMSATS Institute of Information and Technology Abbottabad, operating in the same laboratory. Its total solids (TS) and volatile solids (VS) were 115.6 g/L and 33.61 g/L, respectively, with VS/TS ratio of 0.25. The ABR reactor was fed with synthetic wastewater in order to enrich the sludge and for acclimatization of bacteria to the new substrates.

The reactor was fed on a daily basis with freshly prepared synthetic influent containing NaHCO_3_ (as a source of inorganic carbon for the growth of bacteria), MgCl_2_, KH_2_PO_4_, (1 g/L each), (NH_4_)_2_SO_4_ (0.24 g/L) and trace element solution (1 ml/L). The trace element solution contained Na_2_–EDTA (5 g/L), NaOH (11 g/L), CaCl_2_.2H_2_O (11 g/L), FeCl_2_·4H_2_O (3.58 g/L), MnCl_2_.2H_2_O (2.5 g/l), ZnCl_2_ (1.06 g/L), CoCl_2_.6H_2_O (0.5 g/L), (NH^+^_4_)_6_Mo_7_O_24_.4H_2_O (0.5 g/L) and CuCl_2_.2H_2_O (0.14 g/L). Sucrose is used as source of COD, the starting COD feeding to the ABR reactor was 200 mg/L and, then increased up to 3000 mg/L. The influent was flushed with argon gas for five minutes to create anoxic conditions. The ABR reactor was operated at 20°C, operating at a different HRT (hydraulic retention time) and Organic Loading Rates in order to analyze the process performance.

### 2.3. Experimental Set-up

The lab scale experimental CW consisted of two independent rectangular basins (length: 120 cm, width: 90 cm, depth: 40 cm). The basins were filled with gravel, sand and soil from bottom to top with one layer of each as shown in Figure 2. Each basin had a 10% slope and was equipped with a nozzle outlet to discharge the treated effluent. The CW was planted with *A. donax* (6 shoots/m^2^) taken from the botanical garden of the institute.

An unplanted bed served as a control. Treated effluents were collected directly from the lab scale experimental plant. The operational conditions of the experimental set up of CW are shown in Table 1. All plants, sand and gravel were properly washed before planting into CW system.

Pollutant removal rates (%) were calculated according to the following equation:


*R* (%) = [1–(*Cf*/*Ci*)] × 100 (1)

Where: R is the removal rate, Ci is the concentration (mg/L) of the considered parameter in the untreated WW (influent), Cf is the concentration (mg/L) of the considered parameter in the treatment bed effluent.

### 2.4. Analytical Procedures

Raw and treated samples were analyzed for their BOD, COD, EC, pH, turbidity etc., according to the standard methods [34]. For COD determination closed reflux, colorimetric method included digestion at 150°C for 2 h in COD vials followed by spectrophotometer reading at 530 nm [34]. The pH was measured using a digital pH meter (HANNA, HI 991003 Sensor) while total dissolved solids (TDS) and conductivity were determined by HANNA, HI9835 Microprocessor. Heavy metals were analyzed through atomic absorption spectrophotometer. At least three readings were taken for each parameter each time and then the mean value was calculated.

### 2.5. Statistical Analysis

Collected data were analyzed by descriptive statistics and arithmetic averages of percent removal were calculated using Microsoft Excel XP version 2010 and Origin Lab 8.

## 3. Results and Discussion

### 3.1. Pretreatment of Combined Industrial Wastewater in ABR

The ABR was fed with combined industrial wastewater for treatment at retention time of 12 h. The treated effluent characteristics and percent removal efficiency are showed in Table 2.

The results describe the performance of the ABR for the treatment of combined industrial wastewater, as the concentrations of COD before pretreatment were 70, 189, 284, 379 and 474 mg/L, respectively, for 20, 40, 60 and 80% dilutions, and original wastewater. After pre-treatment with ABR, the COD was reduced to 42, 54, 121, 159, 297 mg/L with 40.0, 40.8, 57.3, 58.0, 37.3% removal efficiency, respectively. The results in Figure 3 show the maximum COD removal efficiency for the 80% dilution of the wastewater through ABR. The ABR also reduced the BOD concentrations of the dilutions in 78 to 82%, as shown in Figure 3. The BOD concentration reduced from 23.3, 25.4, 50.9, 77.0 and 84.8 mg/L to 18.5, 4.16, 5.1, 10.2 and 18.5, respectively. It was observed from the results that ABR showed excellent removal efficiency for BOD.

Total solids were tremendously removed by 84% with the corresponding concentration of 1960 mg/L for original wastewater. The concentration of NO_3_-N was reduced from 24, 59, 83, 98, 145 to 1.8, 6.1, 9.23, 8.9, and 16 mg/L for 20, 40, 60, 80% dilutions and original wastewater. ABR showed 88 to 92% removal efficiency for the NO_3_-Nitrogen as shown in Figure 4.

Similarly, the removal efficiency of NH_4_-N was 87.6, 90.8, 90, 85.9 and 87.8% for the four different dilutions 20, 40, 60, 80% and original wastewater, respectively, as shown in Figure 4. The concentrations of NH_4_- N of 17, 23, 45, 57 and 82 mg/L were reduced to 2.1, 2.1, 4.5, 8 and 10 mg/L. On the other hand, Pb, Ni and Cd removal by reactor was 2.7%, 79% and 85.4% for real industrial wastewater. Heavy metal removal was found in the order Cd > Ni > Pb as shown in Figure 5.

### 3.2. Post Treatment of ABR Effluent with CW

The pre-treated effluent was then further treated by CW for 30 days for each dilution. The results for FWS CW effluent are shown in Figures 6–10 with pollutant removal efficiency.

The results of treatment in CW showed efficient removal efficiency for COD, BOD, TS, nitrates, ammonia and metals like Pb, Ni and Cd. The residual concentrations of COD and BOD were 64.3, 66.7, 67, 76.4, 82.4 and 78.4, 76, 80.3, 80.3 and 78.4 mg/L, respectively, for the corresponding dilutions of 20, 40, 60, 80% and original WW as shown in Figures 6 and 7. The CW showed the highest COD removal efficiency of 82.4% for original WW, but at the same dilution the BOD was reduced to 78.4%. Nitrates and ammonia removal efficiency was found to be 95, 82, 86, 72, 75% for the respective concentrations of 1.8, 6.1, 9.23, 8.9 and 16 mg/L of the corresponding four different dilutions and original pre-treated effluent. Ammonia removal was not satisfactory compared to other parameters and the highest removal efficiency was 70.1% by CW.

### 3.3. Discussion

Previous workers observed that the treatment of complex industrial wastewater reduced the efficiency of the ABR [35], as also observed in the present study. However, ABR showed promising results regarding treatment of BOD in the present work. During anaerobic digestion of organic matter, biochemical reactions take place which are affected by heavy metal presence [35]. It is clear from the results that soluble heavy metals rapidly decreased at the initial concentrations. It depends on which chemical form the heavy metal exists in. The most common and important form of heavy metals are precipitation (as sulfides, carbonates and hydroxides), sorption on to solid form (inhibitory effect of heavy metals on anaerobic sludge) [36]. Ni could be bound in all forms. So it was clear that high initial concentrations were tolerated by the ABR sludge and thus showed the satisfactory removal of heavy metals.

However, the residual concentration of organic (BOD and COD) and heavy metals in the anaerobic reactor effluent usually exceeds the maximum permissible level prescribed by the effluent discharge standards of most developing countries [20, 37-38]. From this standpoint, post-treatment of anaerobic effluent is necessary to reduce these contaminants to the required level [39].

CW normally improves the dissolved oxygen (DO) in wetland. The introduction of excess organic matter may result in depletion of oxygen from an aquatic system. Prolonged exposure to low dissolved oxygen levels (<5.0– 6.0 mg/L) may not directly kill an organism, but will increase its susceptibility to other environmental stresses. Exposure to <30% saturation (<2.0 mg/L oxygen) for one to four days kills most of the biota in a system. If oxygen-requiring organisms perish, the remaining organisms will be air-breathing insects and anaerobic (not requiring oxygen) bacteria [40]. If all oxygen is depleted, aerobic (oxygen-consuming) decomposition ceases. So, treating pollutants in wetlands may help to increase DO, which is consumed by the other aerobes. In this experiment during the post-treatment by CW the DO increased to 8.8 mg/L.

Industrial wastewater was previously treated in two-stage constructed wetland [41] planted with *Typha latifolia* and *Phragmites australis*. For tannery wastewater, CW may be an interesting treatment option. Two-stage series of horizontal subsurface flow CW with *Phragmites australis* (UP series) and *Typha latifolia* (UT series) provided high removal of organics from tannery wastewater, up to 88% of BOD_(5)_ (from an inlet of 420 to 1000 mg/L) and 92% of COD (from an inlet of 808 to 24vx49 mg/L) and of other contaminants, such as nitrogen, operating at hydraulic retention times of 2, 5 and 7 days. Overall mass removals of up to 1294 kg COD/ha/d and 529 kg BOD_(5)_/ha/d were achieved for a loading ranging from 242 to 1925 kg COD/ha/d and from 126 to 900 kg BOD_(5)_/ha/d. Plants were resilient to the conditions imposed, however *P. australis* exceeded *T. latifolia* in terms of propagation [41].

In the present study, *A. donax* was used in the CW for post-treatment, which showed an efficient performance for the further removal of pollutants from the ABR pre-treated effluent. The results confirmed that effluent showed traces of heavy metals Ni and Cd with the corresponding ABR treated wastewater at almost all the levels of dilutions of 20, 40, 60, 80% and original wastewater. CW showed the maximum removal efficiency for Ni and Cd as depicted in Figures 9 and 10, respectively. CW post-treatment of Pb was not satisfactory in reduction of its concentration. Using the San Joaquin Marsh constructed wetlands, the removal efficiencies for four heavy metal elements Cd, Cu, Pb and Zn were evaluated. It was found that the effluent metal concentrations were not substantially lower than the influent. The removal efficiencies of 23.9%, 10.6%, and 17.6% were found for Cd, Cu, and Zn, respectively. No significant reduction was observed for concentrations of Pb [43].

The removal of metals and metalloids from contaminated waters was investigated in constructed wetlands. Metal removal rates in wetlands depend on the type of element (Hg > Mn > Fe ¼ Cd > Pb ¼ Cr > Zn ¼ Cu > Al > Ni > As), their ionic forms, substrate conditions, season, and plant species. Standardized procedures and data are lacking for efficiently comparing properties of plants and substrates. The study depicted the relative treatment efficiency index (RTEI) to quantify treatment impacts on metal removal in constructed wetlands [44].

Various mechanisms, including sedimentation, filtration, chemical precipitation, adsorption, microbial interactions and uptake by vegetation have been attributed with the removal of metals within CW. Specifically, the major processes that are responsible for metal removal in CW are binding to sediments and soils, precipitation as insoluble salts and uptake by plants and bacteria [45]. In CW, substrate interactions remove most metals from contaminated water [46]. The anoxic condition of wetland soil helps create an environment for immobilization of heavy metals in the highly reduced sulfite or metallic form [47]. Wetland plants adsorb and accumulate metals in tissues, which can play important role in CW pollutant treatment efficiency [48]. Phytoremediation, using vegetation to remove, detoxify, or stabilize heavy metal pollutants, is an accepted tool for cleaning polluted soils and waters [49]. Research has also shown that metal storage in sediment is influenced by vegetation. Concentrations of metals were significantly higher in the vegetated sediments than in the non-vegetated sediments [50].

## 4. Conclusion

This paper presented the evaluation results on removal efficiencies for COD, BOD, nitrates, TS, and heavy metals (Cd, Ni, Pb) in ABR and post-treated by a lab scale *Arundo donax* based CW. It was clearly observed that post-treatment accomplished efficient removal of the COD, BOD, TS, Ni, and Cd. The efficiency of both the treatment systems was not very satisfactory for Pb removal. Because of the positive effects of vegetation on metal removal efficiency, CWs containing *A. donax* is recommended for HIE combined wastewater treatment.

## References

1. Q. Mahmood, A. Pervez, B.S. Zeb, H. Zaffar, H. Yaqoob, M. Waseem, Zahidullah, S. Afsheen, “Natural treatment systems as sustainable ecotechnologies for the developing countries”, *Biomed Research International*, vol. 2013, pp. 1-9. 2013

2. B.S. Zeb, Q. Mahmood, A. Pervez, “Characteristics and performance of anaerobic wastewater treatment (a review)”, *Journal of Chemical Society of Pakistan*, vol. 35, pp. 217-232. 2013.

3. A.K. Mungray, Z.V.P. Murthy, J.T. Ashwin, “Post treatment of up- flow anaerobic sludge blanket based sewage treatment plant effluents” A review, *Desalination and Water Treatment*, vol. 22, pp. 220–237.2010

4. A.C. Van Haandel, G. Lettinga, “Anaerobic Sewage Treatment, *A Practical Guide for Regions with a Hot Climate*” John Wiley and Sons, New York. 1994.

5. C.Y. Gomec, “High- rate anaerobic treatment of domestic wastewater at ambient operating temperatures A review on benefits and drawbacks”, *Journal of Environmental Science and Health* , vol. 45, pp. 1169– 84. 2010.

6. A.A. Khan, R.Z. Gaur, V.K. Tyagi, A. Khursheed, B. Lew, A.A. Kazmi, I. Mehrotra, “Sustainable Options of Post Treatment of ABR Effluent Treating Sewage: A Review”, *Resource Conservation and Recycling*, vol. 55, pp. 1232-1251. 2011a.

7. J.Y. Ji, Y.J. Xing, Z.T. Ma, M. Zhang, P. Zheng, “Acute toxicity of pharmaceutical wastewaters containing antibiotics to anaerobic digestion treatment” *Chemosphere*, vol. 91, pp. 1094–1098.2013.

8. B. Lew, M. Belavski, S. Admon, S. Tarre, M. Green, “Temperature effect on ABR reactor operation for domestic wastewater treatment in temperate climate regions”, *Water Science and Technology*, vol. 48, pp. 25–30.2003.

9. G. Lettinga, A.F.M. van Velsen, S.W. Hobma, W. D. Zecuw, A. Klapwijk, “Use of the Upflow Sludge Blanket (USB) Reactor Concept for Biological Wastewater Treatment, Especially for Anaerobic Treatment”, *Biotechnolgy and Bioengineering*, vol. 22, pp. 699-734. 1980.

10. G. Lettinga, A. de Man, A.R.M. van der Last, W. Wiegant, K. van Knippenberg, J. Frijns, J.C.L. van Buuren, “Anaerobic Treatment of Domestic Sewage and Wastewater”, *Water Science and Technology*, vol. 27, pp. 67-73. 1993.

11. L. Seghezzo, R.G. Guerra, S.M. González, A.P. Trupiano,, M.E. Figueroa, C.M. Cuevas, G. Zeeman, G. Lettinga, “Removal Efficiency and Methanogenic Activity Profiles in a Pilot-scale ABR Reactor Treating Settled Sewage at Moderate Temperatures”, *Water Science and Technology*, vol. 45, pp. 243–248.2002.

12. M. von Sperling, C.A.L. Chernicharo, “Biological Wastewater Treatment in Warm Climate Regions”, IWA Publishing London, UK. 2005.

13. G. Lettinga, “Towards feasible and sustainable environmental protection for all”, *Aquatic Ecosystem and Health Manage*ment, vol. 11, pp. 116–24.2008.

14. A. Schellinkhout, C.J. Collazos, “Full- scale Application of the ABR Technology for Sewage Treatment”, *Water Science and Technology*, vol. 25, pp. 159-166. 1992

15. J.T. Souza, E. Foresti, “Domestic Sewage Treatment in an Up- flow Anaerobic Sludge Blanket Sequential Batch System,” *Water Science and Technology*”, vol. l33, pp. 73-84. 1996.

16. A. A Khan, “Post treatment of ABR effluent: Aeration and Variant of ASP”, *PhD Thesis*. IIT Roorkee India. 2012.

17. A.A. Khan, R.Z. Gaur, B. Lew, I. Mehrotra, A.A. Kazmi, “Effect of Aeration on the Quality of Effluent of ABR Reactor Treating Sewage”, *Journal of Environmental Engineering-ASCE*, vol. 137, no 6, pp. 464-472. 2011c.

18. M. A. El-Khateeb, A. Z. El-Bahrawy, “Extensive Post Treatment Using Constructed Wetland”, *Life Science Journal*, vol. 10, pp. 560-568. 2013.

19. W. Verstraete, P. Vandevivere, “New and broader applications of anaerobic digestion”, *Critical Review of Environmental Science and Technology*, vol. 29, pp. 151–173, 1999.

20. N. Sato, T. Okubo, T. Onodera, A. Ohashi, H. Harada, “Prospects for a self - sustainable sewage treatment system: A case study on full - scale ABR system in India's Yamuna River Basin”, *Journal of Environmental Management*, vol. 80, pp. 198–207. 2006.

21. A. K. Mungray, P. Kumar, “Anionic surfactants in treated sewage and sludge: Risk assessment to aquatic and terrestrial environments”, Bioresource Technology, vol. 99, pp. 2919–2929. 2008.

22. M. A El-Khateeb, F. A. El-Gohary, “Combining ABR Technology and Wetland for Domestic Wastewater Reclamation and Reuse, *Water Supply*, vol. 3, pp. 201-208. 2003.

23. B. Hegazy, M. A El-Khateeb, A. El-adly Amira, M. M. Kamel, “Low-cost Wastewater Treatment Technology”, *Journal of Applied Science*, vol. 7, pp. 815-819. 2007.

24. M. A. El-Khateeb, A. Z. Al - Herrawy, M. M. Kamel, F.A. ElGohary, “Use of wetlands as post treatments of anaerobically treated effluent”, *Desalination*, vol. 245, pp. 50–59. 2009.

25. V. Mudgal, N. Madaan, A. Mudgal, “Heavy metals in plants, phytoremediation: Plants used to remediate heavy metal pollution” *Agriculture and Biology Journal of North America1,* vol. 1 , pp. 40- 46. 2010.

26. N. Korboulewsky, R. Wang, V. Baldy, “Purification processes involved in sludge treatment by a vertical flow wetland system: focus on the role of the substrate and plants on N and P removal”, *Bioresource Technology*, vol. 105, pp. 9-14. 2012.

27. C. Wendland, J. Behrendt, T.A. Elmitwalli, I. Al Baz, G. Akcin, O. Alp, R. Otterpohl, “ABR reactor followed by constructed wetland and UV radiation as an appropriate technology for municipal wastewater treatment in Mediterranean countries”, in *Proceedings of the 7th specialized conference on small water and wastewater systems in Mexico,* 2006.

28. P. Worall K. J. Peberdy, M. C. Millett, “Constructed wetland and natural conservation”, *Water Science and Technology*, vol. 3, pp. 205–213. 1997.

29. C. M. Kao, M. J. Wu, “Control of non-point source pollution by a natural wetland”, *Water Science and Technology*, vol. 43, pp. 169- 74. 2001.

30. J. García, P. Aguirre, R. Mujeriego, Y. Huang, L. Ortiz, J. Bayona, “Initial contaminant removal performance factors in horizontal flow reed beds used for treating urban wastewater”, *Water Research*, vol. 38, pp. 1669–1678. 2004.

31. M. von Sperling, C. A. M. Mascarenhas, 2005, “Performance of very shallow ponds treating effluents from ABR reactors”, *Water Science and Technology*, vol. 51(12), 83–90.

32. M. von Sperling, R. K. X. Bastos, M. T. Kato, “Removal of E. coli and helminth eggs in UASB: Polishing pond systems in Brazil”, *Water Science and Technology*, vol. 51, pp. 91-97. 2005.

33. C.A.L Chernicharo, “Post Treatment Options for the Anaerobic Treatment of Domestic Wastewater, A review”, *Environmental Science and Biotechnology*, vol. 5, pp. 73- 92. 2006.

34. American Public Health Association Inc. (APHA), 2005. Standard Methods for the Examination of Water and Wastewater, 21st ed. New York, USA.

35. A. Mudhoo, R. M. Pravish, M. Romeela, “Effects of Microwave Heating on Biogas Production, Chemical Oxygen Demand and Volatile Solids Solubilization of Food Residues. World Academy of Science”, *Engineering and Technology,* vol. 69, pp. 805-810. 2012.

36. M. Sarioglu, A. Serdar, B. Turgay, “Inhibition effects of heavy metals (copper, nickel, zinc, lead) on anaerobic sludge” *Desalination and Water Treatment*, vol. 23, pp. 55-60. 2010.

37. K. J. Prakash, V. K. Tyagi, AA Kazmi, K Arvind, “Post treatment of ABR reactor effluent by coagulation and flocculation process” *Environmental Progress*, vol. 26, pp. 164–168 .2007.

38. I. Machdar, Y. Sekiguchi, A. Sumino, A. Ohashi, H. Harada, “Combination of a ABR reactor and a curtain type DHS reactor as a cost-effective sewage treatment system for developing countries', *Water Science and Technology*, vol. 42, pp. 83–88.2000.

39. T. V. Kumar, A. A. Khan, A. A. Kazmi, I. Mehrotra, A. K. Chopra, “Slow sand filtration of ABR reactor effluent: A promising post treatment technique”, *Desalination*, vol. 249, pp. 571–576.2009.

40. A. M. Gower, “Water quality in catchment ecosystems”, John Wiley & Sons, New York. 1980.

41. C. S. C. Calheiros, A. F. Duque, A. Moura, I. S. Henriques, A. Correia, A. Rangel, P. M. L. Castro, “Substrate effect on bacterial communities from constructed wetlands planted with *Typha latifolia* treating industrial wastewater” *Ecological Engineering*, vol. 35, pp. 744-753.2009.

42. J. T. de Sousa, A. C. van Haandel, A. A. Guimarães, “Post-treatment of anaerobic effluents in constructed wetland systems”, *Water Science and Technology*, vol. 44, pp. 213- 9. 2001.

43. S. Hafeznezami, J.-L. Kim, J. Redman, “Evaluating Removal Efficiency of Heavy Metals in Constructed Wetlands” , *Journal of Environmental Engineering*, vol. 138, pp. 475–482.2012.

44. L. Marchand, M. Mench, D. L. Jacob, M. L. Otte, “Metal and metalloid removal in constructed wetlands, with emphasis on the importance of plants and standardized measurements: A review”, *Environmental Pollution*, vol. 158, pp. 3447-3461.2010.

45. R. H Kadlec, S. D. Wallace, “Treatment wetlands”, 2nd ed. CRC Press/Taylor &Francis Group Boca Raton”, FL. 2008.

46. D. J. Walker, S. Hurl, “The reduction of heavy metals in a storm water wetland”, Ecological Engineering, vol. 18, pp. 407–414. 2002.

47. R. P. Gambrell, “Trace and toxic metals in wetlands, A review”, *Journal of Environmental Quality*, vol. 23, pp. 883–891. 1994.

48. H. Brix, “Functions of macrophytes in constructed wetlands”, *Water Science and Technology* 29: 71-78. 1994.

49. S. Cheng, W. Grosse, F. Karrenbrock, M. Thoennessen, “Efficiency of constructed wetlands in decontamination of water polluted by heavy metals”, *Ecological Engineering* vol. 18, pp. 317–325, 2002.

50. J. H. Choi, S. S. Park, P. R. Jaffé, “The effect of emergent macrophytes on the dynamics of sulfur species and trace metals in wetland sediments”, *Environmental Pollution*, vol. 140, pp. 286–293. 2006.

## Figures and Tables

**Figure 1 fig1:**
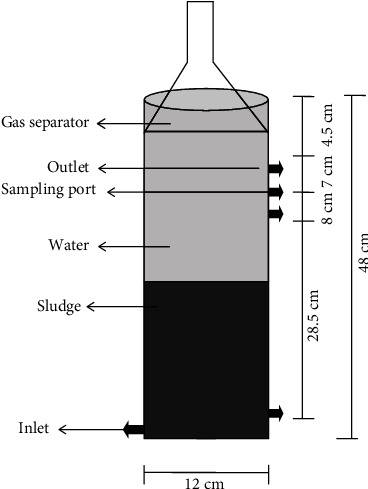
Schematic diagram of a lab scale anaerobic bioreactor with its dimensions.

**Figure 2 fig2:**
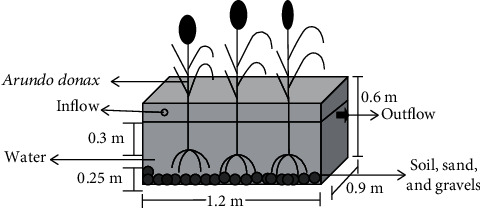
Schematic diagram of a laboratory scale CW for the treatment of combined industrial wastewater.

**Figure 3 fig3:**
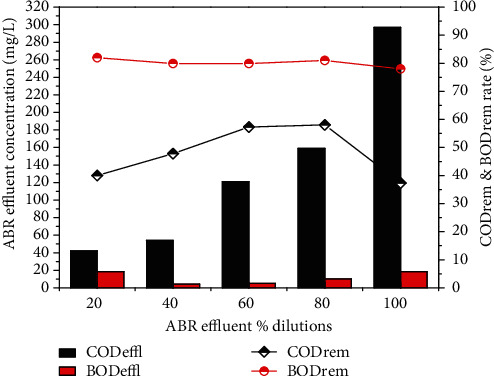
CODeffl and BODeffl concentration (mg/L), CODrem and BODrem efficiency (%) of a lab scale ABR reactor for combined industrial wastewater.

**Figure 4 fig4:**
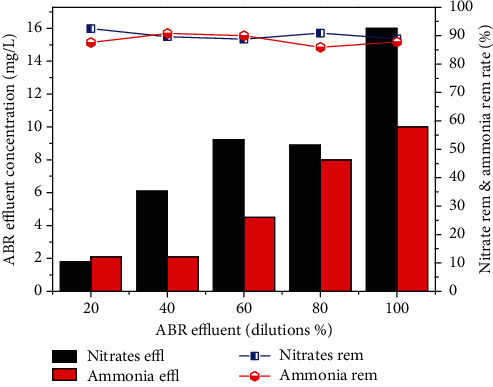
Nitrates Removal efficiency of pretreatment of combined industrial wastewater with ABR.

**Figure 5 fig5:**
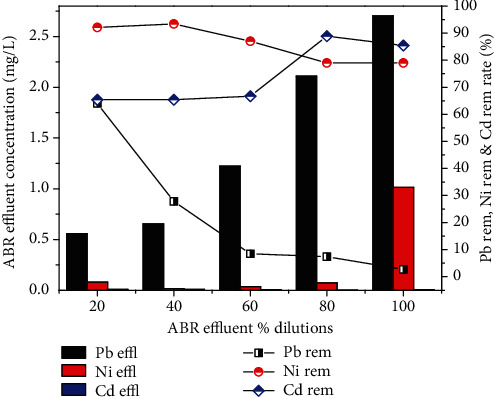
Removal efficiency of Pb, Ni and Cd by ABR.

**Figure 6 fig6:**
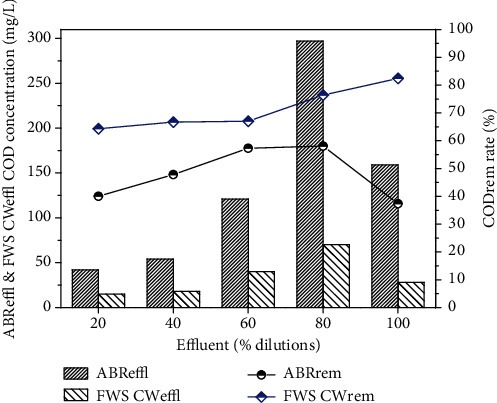
Comparison of ABR and CW for COD removal.

**Figure 7 fig7:**
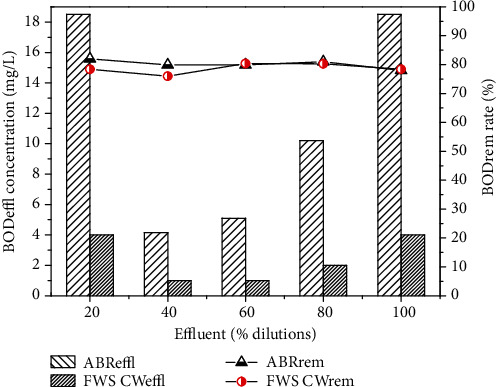
Comparison of ABR and CW for BOD removal.

**Figure 8 fig8:**
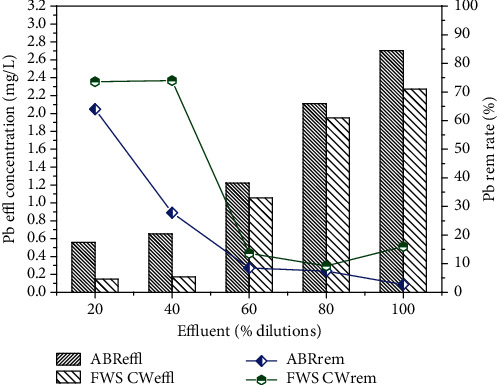
Comparison of % removal efficiency of Pb in ABR and FWS CW.

**Figure 9 fig9:**
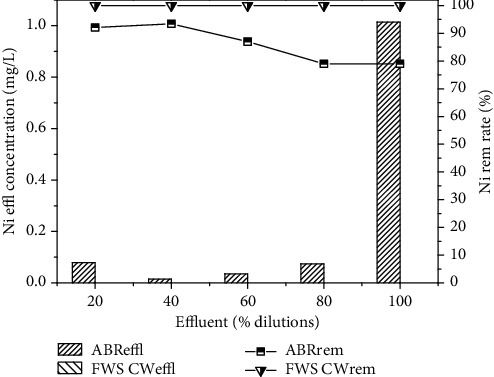
Comparison of percent removal efficiency of Ni in ABR and FWS SCW.

**Figure 10 fig10:**
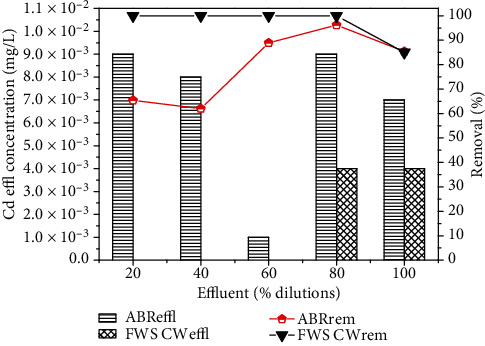
Comparison of % removal efficiency of Cd in ABR and FWS CW.

**Table 1 tab1:** Dimensions and operating conditions of experimental CW.

Dimensions of CW	
Length	1.2 m
Width	0.9 m
Total height	0.6 m
Total container volume	0.432 m^3^
Water depth	0.3 m
Substrate depth	0.25 m
Plant name	*Arundo donax* L (Giant reed)
No. of rhizome/m^2^	3

Operational conditions	
OLR	538 kg/ha/d
HRT	3 days
Hydraulic load rate (HLR)	862 m^3^/ha/d

**Table 2 tab2:** The performance of ABR in treating combined industrial effluents.

Parameters	Influent/raw wastewater					ABR effluent					% removal				
	20%	40%	60%	80%	Original WW	20%	40%	60%	80%	Original WW	20%	40%	60%	80%	Original WW
pH	8.1	8.48	8.6	8.76	10.2	7.89	8.31	8.52	8.66	8.41	—	—	—	—	—
Conductivity (*μ*s)	627	646	645	676	702	654	616	645	687	702	—	—	—	—	—
TDS	378	330	344	394	411	339	324	334	357	365	10	2	3	9	11
VS	712	812	780	1333	1400	83	85	93	215	115	88	89.5	88	83.8	91.7
TS	600	520	1050	1160	1960	45	86.3	163.9	97.2	305	92	83.5	86	90	84
COD	70	189	284	379	474	42	54	121	159	297	40	47.8	57.3	58.04	37.34
BOD	23.3	25.4	50.9	77	84.8	18.5	4.16	5.1	10.2	18.5	82	80	80	81	78
Nitrates	24	59	83	98	145	1.8	6.1	9.23	8.9	16	92.5	89.6	88.8	90.9	88.9
Ammonia	17	23	45	57	82	2.1	2.1	4.5	8	10	87.6	90.8	90	85.9	87.8
Lead	0.131	0.653	1.335	1.851	2.337	0.558	0.653	1.222	2.109	2.703	64	27.8	8.46	7.4	2.7
Nickle	.117	.225	.315	.271	.403	0.079	0.015	0.035	0.074	1.014	92.1	93.4	87	79	79
Cadmium	.026	.026	.021	.009	.048	0.009	0.009	0.007	0.001	0.007	65.4	65.4	66.7	88.9	85.4
